# Comparing the estimates of effect obtained from statistical causal inference methods: An example using bovine respiratory disease in feedlot cattle

**DOI:** 10.1371/journal.pone.0233960

**Published:** 2020-06-25

**Authors:** Ju Ji, Chong Wang, Zhulin He, Karen E. Hay, Tamsin S. Barnes, Annette M. O’Connor

**Affiliations:** 1 Department of Statistics, Iowa State University, Ames, Iowa, United States of America; 2 QIMR Berghofer Medical Research Institute, Herston, Australia; 3 The University of Queensland, School of Veterinary Science, Gatton, Queensland, Australia; 4 The University of Queensland, Queensland Alliance for Agriculture and Food Innovation, Gatton, Queensland, Australia; 5 Department of Veterinary Diagnostic and Production Animal Medicine, Iowa State University, Ames, Iowa, United States of America; 6 Department of Large Animal Clinical Sciences, Michigan State University, East Lansing, Michigan, United States of America; National Veterinary School of Toulouse, FRANCE

## Abstract

The causal effect of an exposure on an outcome of interest in an observational study cannot be estimated directly if the confounding variables are not controlled. Many approaches are available for estimating the causal effect of an exposure. In this manuscript, we demonstrate the advantages associated with using inverse probability weighting (IPW) and doubly robust estimation of the odds ratio in terms of reduced bias. IPW approach can be used to adjust for confounding variables and provide unbiased estimates of the exposure’s causal effect. For cluster-structured data, as is common in animal populations, inverse conditional probability weighting (ICPW) approach can provide a robust estimation of the causal effect. Doubly robust estimation can provide a robust method even when the specification of the model form is uncertain. In this paper, the usage of IPW, ICPW, and doubly robust approaches are illustrated with a subset of data with complete covariates from the Australian-based National Bovine Respiratory Disease Initiative as well as simulated data. We evaluate the causal effect of prior bovine viral diarrhea exposure on bovine respiratory disease in feedlot cattle. The results show that the IPW, ICPW and doubly robust approaches would provide a more accurate estimation of the exposure effect than the traditional outcome regression model, and doubly robust approaches are the most preferable overall.

## Introduction

In veterinary science, the goal of many observational studies is to estimate the causal effect of exposures on disease outcomes. There are many approaches to estimation. In general, methods used to adjust for confounding in observational studies can be classified into two categories: G-methods and stratification-based methods. G-methods include IPW, standardization and G-estimation, where the conditional exchangeability has been used in subsets defined by covariates to estimate the causal effect of exposures on outcomes in the entire population (marginal). Stratification-based methods include stratification, restriction and matching, but the conditional exchangeability is used in subsets defined by covariates to estimate the association between exposures and outcomes in those subsets only (conditional) [1]. The commonly used outcome regression models belong to the stratification-based category. Therefore, in this manuscript, we illustrate the advantages of methods from G-methods category to illustrate the advantages to the estimation of the average causal effect and contrast them with more commonly used outcome regression models that are likely more recognizable to veterinary researchers. Specifically, we only focus on three methods to estimate the average causal effect: IPW, inverse conditional probability weighting (ICPW) and the doubly robust approach, and we refer them as the causal inference estimation approaches throughout the paper. The rationale for this paper is to introduce causal inference estimation approaches to veterinary researchers using realistic example data and to illustrate the advantages (reduced bias in the estimation of the average causal effect) when compared to traditional outcome regression model-based approaches to estimation.

In this paper, the advantages of causal inference methods will be illustrated by showing their ability to provide an unbiased estimation of the average causal effect. Because of our goal to illustrate the advantages of causal inference approaches, the target reader for the paper is a quantitative epidemiologist comfortable with regression modeling approaches, estimation approaches, matrix algebra, and reading mathematical formulas. We have provided more statistical detail than most veterinary methods manuscripts and less than most statistical methodology papers. This paper is not intended as either a step-by-step tutorial for causal inference methods nor a treatise on causal inference methods. We provide appropriate references throughout for readers who want more in-depth knowledge.

There is a need for an illustrative example with causal inference methods because, although causal inference approaches have been available for a long time [2], adoption of the methods in veterinary epidemiology appears to lag behind other epidemiology disciplines. For example, in 2018 the American Journal of Epidemiology published 344 articles, of which 14 referred to logistic regression (an outcome regression model approach) and 18 referred to inverse probability weighting or propensity scoring in the title or abstract. By contrast in 2018, Preventive Veterinary Medicine published 268 articles, of which 31 referred to logistic regression and one referred to inverse probability weighting or propensity scoring in the title or abstract (see Appendix for exact search string). These statistics are an imperfect measure of uptake of the methods, but they are likely reflective of the differences in uptake.

This paper is organized as follows: Section 2 briefly recaps the main concepts of marginal models and estimation of average causal effects using an example based on infectious causes of Bovine Respiratory Disease (BRD). The BRD example is carried throughout the paper and was chosen because it is a common topic of research in veterinary science. As respiratory disease is common in all species, the topic provides a relatable example for many veterinary epidemiologists, even those not working in bovine production. We purposefully selected a subset of data with complete covariates for our example. This subset of data is not necessarily representative of the original dataset. For a thorough analysis of the original data and the associations found in the dataset, we direct the readers to [3–5]. Section 3 outlines in detail each of the approaches, models used in the analysis, and the estimation equations. Aspects of Section 3 require an understanding of matrix algebra notation. This section is intended to set the stage for the later sections by introducing the models and estimation equations to be used. Readers who are unfamiliar with matrix algebra or who already know the basics for the estimation methods can skip Section 3. Section 4 reports the results of the approaches applied to realistic BRD data and simulation studies which document the advantages of causal inference methods in reducing the biases in estimation. Comparisons among the approaches are discussed in Section 5. Section 6 concludes with a summary and further discussion.

## Introduction to data and marginal causal effects

BRD is an important economic disease that causes morbidity and mortality in feedlot cattle; it is also a major contributor to antibiotic use in cattle production. In feedlot cattle, BRD usually occurs soon after cattle have arrived at the feedlot due to increased stress associated with transportation, mixing of the cattle, a new feed ration, and exposure to multiple pathogens. The disease is multifactorial in origin with many factors contributing to increased risk. Previous studies have shown that serologically negative animals exposed to Bovine Viral Diarrhea Virus (BVDV) at the feedlot are at increased risk of developing BRD [3, 6]. Exposure to BVDV before arrival at the feedlot, either by vaccination or natural exposure, could offer cattle protection against BRD [4, 7]. However, there are some individual-level risk factors and unmeasured feedlot-level risk factors that have direct effects on both prior BVDV exposure and BRD incidence, which makes the evaluation of the direct effect of prior BVDV exposure on BRD difficult. Commonly, we refer to this situation as confounding. Hernan and Robins defined the confounding in chapter 7 of Causal Inference as the bias that results from the presence of causes shared by treatment (exposure) and outcome [15]. However, the direct effect of BVDV exposure prior to arrival at the feedlot on BRD incidence is difficult to evaluate because the relationship is confounded by individual-level risk factors and unmeasured feedlot-level risk factors. Knowing the effect of prior BVDV exposure is of particular interest because BVDV exposure can be manipulated, especially with vaccination, and therefore this represents a potential intervention point for preventing and reducing BRD. Other BRD risk factors, such as age, weight, and breed, are less amenable or impossible to manipulate.

Given that both outcome (BRD) and exposure (prior BVDV exposure) are binary, the average causal effect of the exposure can be evaluated through the odds ratio. In a cohort study, because the disease event is incident, the odds ratio is more appropriately defined as the disease odds ratio. There are two types of odds ratios estimates, conditional or marginal (unconditional), depending on whether we are conditioning on, or marginalizing over the confounding covariates. A conditional odds ratio can help to decide whether an exposure is beneficial for an animal with particular characteristics, while a marginal odds ratio can be used to assess the effect of the exposure in the population as a whole. Veterinary epidemiologists often fit an logistic regression and obtain the estimates of the coefficient (*β*) of the exposure and use the estimates of *exp*(*β*) as the odds ratio, and for a model with covariates, this represents a conditional rather than a marginal estimator. Collapsing over the other covariates, the marginal odds ratio can be different from the conditional odds ratio. This is called the non-collapsibility of the odds ratio. For more explanation and examples of the non-collapsibility, please see Greenland and Robins 2009 [8] and Hernan 2011 [9]. The causal inference approaches enable estimation of the marginal causal effects, which correspond with the traditional parameters of interest in randomized trials [10–12]. The marginal risk can then be used to estimate relative measures such as the risk ratio or the odds ratio.

Therefore, our goal is to illustrate approaches to estimating the marginal odds ratio with and without the exposure to BVDV prior to arrival at the feedlot to BVDV on the outcome, BRD. Exposure to BVDV prior to arrival at the feedlot was measured by the presence of antibodies to BVDV in blood samples collected when the animals arrived at the feedlot. We used an independently developed, a priori causal diagram based on biologically plausible pathways to inform the covariates to include in the estimation of the exposure effect [5]. We determined the minimal sufficient set of fixed effects required to assess the total effect of prior exposure to BVDV (as indicated by the presence of antibodies to BVDV at arrival) on BRD outcome [13, 14]. In addition, given the importance of cluster variables in veterinary settings, we explicitly included feedlot in the model to enable assessment of the difference in the three methods when clustering is present [15].

## Materials and methods

### Notations

Throughout this paper, we use bold letters such as ***A*** and ***β*** to denote vectors. We use non-bold letters such as *A* and *β* to denote scalar quantities. Let *Y*_*ij*_ be the dichotomous observable outcome for the *j*^*th*^ individual (*j* = 1, 2, …, *n*_*i*_) in the *i*^*th*^ cluster (*i* = 1, 2, …, *I*). *n*_*i*_ is the number of individuals in each cluster. *I* is the total number of clusters. *A*_*ij*_ is a binary variable of the individual-level exposure (*A*_*ij*_ = 1 if exposed and *A*_*ij*_ = 0 if unexposed). Let ***X***_*ij*_ = (*X*_*ij*,1_, *X*_*ij*,2_, …, *X*_*ij*,*k*_) be a k-dimensional vector of individual-level covariates, which may confound the relationship between exposure and outcome. Each individual has two potential outcomes: $Y_{ij}^{0}$ and $Y_{ij}^{1}$. If the individual was actually exposed, we can only observe $Y_{ij}^{1}$ but not $Y_{ij}^{0}$. Otherwise if the individual was not exposed, we can only observe $Y_{ij}^{0}$ but not $Y_{ij}^{1}$. The connection between the observable outcome and the potential outcomes is $Y_{ij}=A_{ij}Y_{ij}^{1}+\left(1-A_{ij}\right)Y_{ij}^{0}$. The concept of potential outcomes was introduced in the context of both randomized and non-randomized studies by Rubin [16].

Given the individuals are nested in clusters, we can use a vector to represent the outcome in the *i*^*th*^ cluster, $Y_{i}={\left(Y_{i1},Y_{i2},...Y_{in_{i}}\right)}^{\prime }$, in which each element is the observable outcome for each individual within cluster *i*. Similarly, the exposure and covariates in the *i*^*th*^ cluster are denoted as vectors $A_{i}={\left(A_{i1},A_{i2},...A_{in_{i}}\right)}^{\prime }$ and $X_{i}={\left(X_{i1},X_{i2},...,X_{in_{i}}\right)}^{\prime }$. The cluster-level potential outcomes vectors are $Y_{i}^{0}={\left(Y_{i1}^{0},Y_{i2}^{0},...,Y_{in_{i}}^{0}\right)}^{\prime }$ and $Y_{i}^{1}={\left(Y_{i1}^{1},Y_{i2}^{1},...,Y_{in_{i}}^{1}\right)}^{\prime }$. In later sections, we will fit an exposure model with ***A***_*i*_ as the response and an outcome model with ***Y***_*i*_ as the response. *U*_*iA*_ and *U*_*iY*_ represent the random cluster effects in the exposure model and the outcome model respectively.

### Approaches overview

In total, we use six approaches for estimation of the odds ratio. The approaches are summarized here and described in more detail below.

1. A univariate outcome model (UOM) approach where only the exposure of interest is regressed on the outcome, and feedlot is the random effect.
2. A multivariate outcome model (MOM) approach where the exposure of interest and all potential individual-level confounding variables identified in the minimum sufficient set are regressed on the outcome, and feedlot is the random effect. [13].
3. An inverse probability weighting (IPW) approach, with an exposure model that uses logistic regression with feedlot as the random effect to estimate the probability of exposure (propensity score) for each individual. The inverse of the probability of exposure is then used to weight each individual in the estimation of the marginal odds ratio.
4. A modified version of the IPW approach that addresses concerns about unmeasured cluster-level confounders called inverse conditional probability weighting (ICPW), which is used to deal with cluster effect by stratification. For this approach, the exposure model uses a conditional logistic regression to estimate the probability of exposure for each individual; however, the odds ratio estimated from the outcome model is still the marginal odds ratio.
5. IPW with a doubly robust estimation approach. In section 2.7 we provide more detail about the rationale incorporating the doubly robust estimation.
6. ICPW with a doubly robust estimation approach.

### Outcome model

The outcome model has a binary response, and the exposure alone or together with the covariates are the explanatory variables with either binary or continuous form. The set of covariates are technically all the potential individual-level confounders required to provide an unbiased estimate of the exposure-outcome relationship. We make this assumption about the covariates but we will not repeat it later in the text. Further, for the BRD dataset used in our application has a clustered structure due to multiple feedlots, the most common way of analysis is to fit a generalized linear mixed model with logit link and a random effect for the cluster variable as follows:

$$\begin{array}{c} p\left(Y_{ij}=1|A_{ij},X_{ij},U_{iY};\beta \right)=p_{Y,ij},\\ log(\frac{p_{Y,ij}}{1-p_{Y,ij}})=\beta_{a}A_{ij}+\sum_{p=1}^{k}\beta_{p}X_{ij,p}+U_{iY}, \end{array}$$ (1)

where ***β*** = (*β*_*a*_, *β*_1_, *β*_2_, …, *β*_*k*_)′ are the effect of the explanatory variables on the outcome on logit scale, and $U_{iY}∼N\left(0,\sigma_{Y}^{2}\right)$ is the random cluster effect.

The estimator of the effect of exposure on the outcome is based on the risk in each exposure group calculated as $expit(\beta_{a}+\sum_{p=1}^{k}\beta_{p}X_{ij,p})$ for *A*_*ij*_ = 1 and $expit(\sum_{p=1}^{k}\beta_{p}X_{ij,p})$ for *A*_*ij*_ = 0, where the “*expit*()” is defined as $expit\left(z\right)=\frac{exp(z)}{1+exp(z)}$ for any variable *z*. The “*expit*()” is also called the inverse logit function. Note that this estimate is different from the routine *exp*(*β*) estimator employed very frequently, which is an estimate of the conditional effect of exposure on the outcome.

### Exposure model

The exposure model is fitted to estimate the probability of exposure given the covariates, which is also called the propensity score [17]. It is for this reason we refer to this as the exposure model. The estimated probability of exposure will be used to compute the inverse probability weights for each individual. Similar to the outcome model, we also fit a generalized linear mixed model with logit link and a random cluster effect:

$$\begin{array}{c} P\left(A_{ij}=1|X_{ij},U_{iA};\alpha \right)=p_{A,ij},\\ log(\frac{p_{A,ij}}{1-p_{A,ij}})=\sum_{p=1}^{k}\alpha_{p}X_{ij,p}+U_{iA}, \end{array}$$ (2)

where ***α*** = (*α*_1_, *α*_2_, …, *α*_*k*_)′ are the effects of the covariates on the exposure of interest on logit scale, and $U_{iA}∼N\left(0,\sigma_{A}^{2}\right)$ is the random cluster effect. From this model we obtain the estimated probability of exposure as the second line of Eq 2, which will be used to weight each individual before estimating the marginal odds ratio.

### Assumptions and estimates for IPW approach

Here we very briefly present the formula and assumptions for the IPW estimator. For a more through description of the assumptions and formulas of IPW, readers are referred to Chapters 2 and 3 in Causal Inference by Hernan and Robins [1].

There are three assumptions required in order to obtain an unbiased IPW estimator [1]:

1. *Consistency*: $Y_{ij}=Y_{ij}^{a}I\left(A_{ij}=a\right)$ *for all i* and *j*, which means that an individual with observed exposure *A* equal to *a*, has observed outcome *Y* equal to its potential outcome *Y*^*a*^.
2. *Conditional exchangeability*: $\left\{Y_{ij}^{0},Y_{ij}^{1}\right\}∐A_{ij}|X_{ij},U_{iA}$ *for all i and j*, which means that the risk of the outcome under the potential exposure level *a* among the exposed is the same as the risk under the potential exposure level *a* among the unexposed.
3. *Positivity: For all i*, $\sum_{j=1}^{n_{i}}a_{ij}\neq 0$ *or n_i_, and 0 < P(A_ij_ = a_ij_|**X**_i_, U_iA_)<1 for all i and j, where a_ij_ = 0 or 1*, which means that the conditional probability of being under every exposure level is greater than 0 and less than 1.

Given the validity of these assumptions in the dataset, the IPW estimator gives the average causal effect of exposure on the outcome by creating a pseudo-population by weighting each individual according to the inverse of the probability of exposure. In the pseudo-population the exposure and the measured confounders are independent. In lay terms, the IPW estimator provides an estimate of the risk of the outcome in each exposure group. This is achieved because the population is balanced with respect to confounders due to weighting by the inverse probability of exposure [18]. More formally, this can be summarized as follows. The risk of the outcome in the exposure group *A* = *a* is estimated by the inverse probability of the exposure weighted mean of the outcome Y when exposure *A* = *a* is given. The risk of disease under exposure is estimated by ${\hat{P}}_{1,IPW}$ and the risk of disease under no exposure is given by ${\hat{P}}_{0,IPW}$, which can be expressed as the Eq 3.

$$\begin{array}{cc} {\hat{P}}_{1,IPW} & =\frac{1}{N}\sum_{i=1}^{I}\sum_{j=1}^{n_{i}}\frac{A_{ij}Y_{ij}}{P(A_{ij}=1|X_{ij},{\hat{U}}_{iA};\hat{\alpha })},\\ {\hat{P}}_{0,IPW} & =\frac{1}{N}\sum_{i=1}^{I}\sum_{j=1}^{n_{i}}\frac{\left(1-A_{ij}\right)Y_{ij}}{1-P(A_{ij}=1|X_{ij},{\hat{U}}_{iA};\hat{\alpha })}, \end{array}$$ (3)

where the weights for the IP estimator on the denominator, $P(A_{ij}=1|X_{ij},{\hat{U}}_{iA};\hat{\alpha })$, are obtained with the random cluster effect exposure model in Eq 2. In the case where the weights have extreme value, IPW may lead to biased estimate of the causal effect. Instead, stabilized IPW have been proposed to tackle these extreme values and provide unbiased estimation [19]. In the BRD dataset used in our application, the range of the estimated conditional probabilities of exposure to BVDV is from 0.19 to 0.98, thus using IPW is sufficient. As can be seen in the equation above, the IP weights are in the denominator of the estimator formula, hence the term inverse probability weighting. It is this process that creates the pseudo-population that balances the confounder distribution across the level of the exposure variable A, makes the confounders and the exposure independent and enables estimation of the average causal effect [17]. The average causal effect of the binary exposure *A*_*ij*_ on the binary outcome of interest Y can be estimated using the odds ratio as $\frac{{\hat{P}}_{1,IPW}/\left(1-{\hat{P}}_{1,IPW}\right)}{{\hat{P}}_{0,IPW}/\left(1-{\hat{P}}_{0,IPW}\right)}$. If the estimated odds ratio covers 1 (i.e. ${\hat{P}}_{1,IPW}={\hat{P}}_{0,IPW}$), it suggests that exposure *A* does not have an average causal effect on outcome *Y* in the population [1]. One potential limitation of IPW approach is that there should be no unmeasured confounders, including the cluster-level confounding variables. However, the inverse conditional probability weighing (ICPW) can provide a robust estimation for a case with unmeasured cluster-level confounders.

### Inverse conditional probability weighting (ICPW)

In veterinary populations, clustering is common and the ability to adjust for cluster-level variables is limited; therefore this condition may not be guaranteed. For example, in the cattle population such as we use for the BRD example, an unmeasured cluster-specific factor such as surrounding environment of the farm, which likely impacts the occurrence of BRD (the outcome Y), is also likely associated with the covariates such as animals’ health status. Inclusion of the cluster-level variable as a random effect may not be sufficient to control for the confounding effect. In this circumstance, the inverse conditional probability weighting (ICPW) approach proposed by He [20] can solve this issue by using the sufficient statistic for the unmeasured cluster-level confounder (denoted as *V*_*i*_). For extensive discussions about either the concept of sufficient statistics or the ICPW, we refer the reader to other literature [20, 21]. However, for completeness, we present the assumption and formula and briefly discuss the difference from the IPW estimator in the supporting information section.

In the ICPW approach, we fit a conditional logistic regression as the exposure model (conditional on *V*_*i*_, which is the cluster-specific variable). This approach treats the cluster as a stratifying variable rather than a random effect, and we obtain an estimate of the exposure probability conditional on the cluster. The exchangeability and positivity assumptions mentioned in Section 2.4 need to be modified due to the usage of the sufficient statistic of *V*_*i*_, and an assumption regarding existence of the sufficient statistic is also required. More details are provided in the supporting information.

The major difference between the IPW and ICPW is that we construct the probability of exposure (the propensity score) *A*_*ij*_ conditional on individual-level covariates ***X***_*ij*_ and the sufficient statistic of the cluster-level covariates *V*_*i*_ (denoted as *S*_*i*_ = *S*(***A***_*i*_)), which is a function of $A_{i}=\left(A_{i1},A_{i2},...,A_{in_{i}}\right)$. This is done using a conditional logistic regression for the exposure model. Formally, this is as follows in Eq 4, let ***a***_*i*_ be the observed value of ***A***_*i*_, the exposure model for ICPW estimator can be fitted with a conditional logistic regression for all individual *j* in cluster *i* as follows:

$$\begin{array}{cc} P(A_{ij}=a_{ij}|X_{ij},S_{i};\alpha ) & =\frac{P(A_{ij}=a_{ij},S_{i}=S\left(a_{i}\right)|X_{ij},V_{i};\alpha )}{P(S_{i}=S\left(a_{i}\right)|X_{ij},V_{i};\alpha )} \end{array}$$ (4)

For the ICPW estimator, we use ${\hat{P}}_{a,ICPW}$ in Eq 5 to estimate the risk of disease under exposure level *A* = *a*. For a binary exposure, *A*_*ij*_ the estimators are as follows:

$$\begin{array}{cc} {\hat{P}}_{1,ICPW} & =\frac{1}{N}\sum_{i=1}^{I}\sum_{j=1}^{n_{i}}\frac{A_{ij}Y_{ij}}{P(A_{ij}=1|X_{ij},S_{i};\hat{\alpha })},\\ {\hat{P}}_{0,ICPW} & =\frac{1}{N}\sum_{i=1}^{I}\sum_{j=1}^{n_{i}}\frac{\left(1-A_{ij}\right)Y_{ij}}{1-P(A_{ij}=1|X_{ij},S_{i};\hat{\alpha })}. \end{array}$$ (5)

Let $\hat{\alpha }$ be the conditional maximum likelihood estimator that maximizes the joint conditional likelihood.

### Doubly robust

The doubly robust approach has two modeling components and produces a consistent effect estimator if at least one of the two component models is correctly specified and assuming that there are no unmeasured confounders. In other words, it gives us a second chance to correctly specify at least one of the models. The first component is the exposure model, which could either be a random effect logistic regression model as Eq 2 or a conditional logistic regression model as Eq 4. The second component is the outcome model in Eq 1. The formula for the doubly robust estimator is provided by Cao et al. [22]. We use ${\hat{P}}_{a,DR}$ to represent the doubly robust estimators for the risk of disease under exposure *A* = *a*. For the binary exposure variable *A*_*ij*_, the estimators are as follows:

$$\begin{array}{cc} {\hat{P}}_{0,DR} & =\frac{1}{N}\sum_{i=1}^{I}\sum_{j=1}^{n_{i}}[\frac{1-I(A_{ij}=1)}{1-PS}Y_{ij}+\frac{I(A_{ij}=1)-PS}{1-PS}m_{0}\left(X_{ij},{\hat{U}}_{iY};\hat{\beta }\right)],\\ {\hat{P}}_{1,DR} & =\frac{1}{N}\sum_{i=1}^{I}\sum_{j=1}^{n_{i}}[\frac{I(A_{ij}=1)}{PS}Y_{ij}-\frac{I(A_{ij}=1)-PS}{PS}m_{1}\left(X_{ij},{\hat{U}}_{iY};\hat{\beta }\right)], \end{array}$$ (6)

where “PS” is the probability of exposure (propensity score) obtained from the exposure model. *m*_0_, *m*_1_ are the risks of the outcome in each exposure group obtained from the outcome model.

For the IPW with a doubly robust estimation approach, the “PS” part is $P(A_{ij}=1|X_{ij},{\hat{U}}_{iA};\hat{\alpha })$ for a random cluster effect exposure model shown as Eq 2. For the ICPW with a doubly robust estimation approach, the “PS” part is the conditional logistic regression model $P(A_{ij}=1|X_{ij},S_{i};\hat{\alpha })$ shown as Eq 4. For the outcome model part,

$$\begin{array}{cc} m_{0}\left(X_{ij},{\hat{U}}_{iY};\hat{\beta }\right) & =expit(\sum_{p=1}^{k}{\hat{\beta }}_{p}X_{ij,p}+{\hat{U}}_{iY})\\ m_{1}\left(X_{ij},{\hat{U}}_{iY};\hat{\beta }\right) & =expit({\hat{\beta }}_{a}+\sum_{p=1}^{k}{\hat{\beta }}_{p}X_{ij,p}+{\hat{U}}_{iY}), \end{array}$$

where $m_{0}\left(X_{ij},{\hat{U}}_{iY};\hat{\beta }\right)$ is the estimated risk of the disease outcome under no exposure, and $m_{1}\left(X_{ij},{\hat{U}}_{iY};\hat{\beta }\right)$ is the estimated risk of the disease outcome under exposure, which was shown as Eq 1.

## Results

### Infectious causes of bovine respiratory disease data application

**Fig 1:**
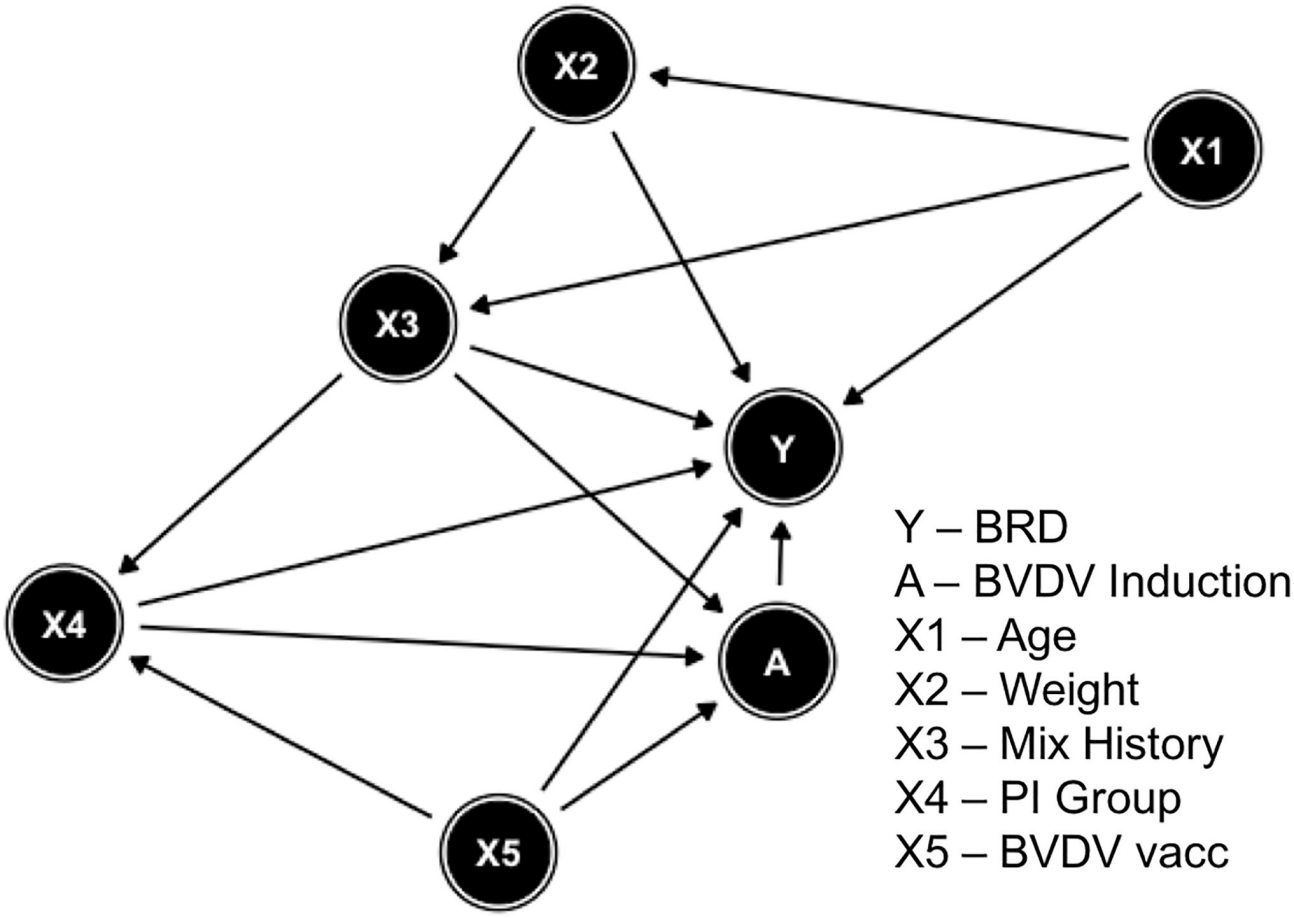
Directed acyclic graph (DAG) of the relationship among outcome, exposure and confounding variables.

In the Australian-based National Bovine Respiratory Disease Initiative (NBRDI) data, BRD is the outcome and BVDV serology upon arrival at the feedlot is the exposure of interest (BVDV induction). Age, weight, mix history, persistently infected (PI) group, and BVDV vaccination are the five individual-level covariates, which may confound the relationship between BVDV induction and BRD. Feedlot is the cluster identifier. Fig 1 presents the relationship among all the variables through a directed acyclic graph (DAG). A detailed description for this dataset and the proposed DAG can be found in Chapter 4 and Chapter 11 in Hay 2015 [5]. The original NBRDI data has 35,131 animals with the BRD incidence rate as 0.176. After matching data from vendor questionnaire with serology results using animal identifier, the sample size was reduced to 2,272 with BRD incidence rate increased to 0.528. Observations with missing values in variables age, mix history, and BVDV vaccination were deleted. We also deleted observations from one feedlot with only two animals, which routinely backgrounded small groups of animals for extended periods. The final subset of data that we use has 1,552 animals nested within 10 feedlots. The BRD incidence in our final dataset is 0.537 which is higher than the prevalence from the original NBRDI. As mentioned, the association observed in the complete NBRDI data has been described extensively previously [3–5]; therefore, our focus is not an extensive reanalysis of the data or making any clinical inference but rather as a demonstration. To distinguish the subset we use from the NBRDI data, we refer to our dataset as the Subset-BRD dataset. Table 1 includes the detailed information of the variables we used in the application. Table 2 shows the descriptive statistics for the Subset-BRD dataset within each category of prior BVDV exposed and non-exposed groups, where means and standard deviations are reported for continuous variables and proportions are reported for categorical variables. Table 3 shows the estimated parameters in the outcome model and the random effect exposure model for our subset of 1,552 study subjects.

**Table 1:** Table of variables from NBRDI data.

Variable	Variable name	Interpretation
*Y*	**Bovine Respiratory Disease (BRD)**	
	no	No BRD diagnosed between day 0 ^a^ and day 50
	yes	BRD diagnosed between day 0 and day 50
*A*	**Bovine Viral Diarrhea Virus (BVDV) induction**	
	no	Seronegative to BVDV at induction, no BVDV exposure prior to induction at the feedlot^b^
	yes	Seropositive to BVDV at induction, BVDV exposure prior to arrival at the feedlot
*X*1	**Age**	Age at induction at the feedlot
*X*2	**Weight**	Weight at induction at the feedlot
*X*3	**Mix History (collapsed version)**	
	no, high	No mixing before day -27 and ≥4 groups^c^ defined on day -28 forming cohort
	no, low	No mixing before day -27 and <4 groups defined on day -28 forming cohort
	yes, high	Mixing before day -27 and ≥4 groups defined on day -28 forming cohort
	yes, low	Mixing before day -27 and <4 groups defined on day -28 forming cohort
*X*4	**Persistently Infected (PI) Group**	
	no BVDV in cohort^d^	No BVDV in the cohort
	PI animal before cohort	PI animals in the groups defined on day -28 and in cohort
	PI animal in cohort	PI animals in the cohort
	TI but no PI in cohort	Only transiently infected animals in the cohort
*X*5	**BVDV vaccination**	
	no	No Pestigard^™^ vaccine administered prior to day -14
	yes	Pestigard^™^ vaccine administered prior to day -14
*U*_*i*_	**Feedlot**	Feedlot Identifier

^a^ Day 0 is the date of induction (processing) which was on or close to the day of arrival at the feedlot; day -27 is 27 days before day 0

^b^ Feedlot is an intensive commercial unit in a single location involved in the finishing stages of beef production

^c^ Group is an identifier used for animals within a cohort that were together on a given day prior to induction

^d^ Cohort comprises all study animals grouped together in a pen following their animal-level induction, and cohorts were nested within feedlots

**Table 2:** Descriptive table for Subset-BRD dataset.

Variable	Variable name	*A* = 1 (BVDV induction)	*A* = 0 (no BVDV induction)
*X*1	**Age**	1.91 (0.441) ^a^	1.92 (0.377)
*X*2	**Weight**	4.38 (0.369)	4.37 (0.353)
*X*3	**Mix History (collapsed version)**		
	no, high	0.387 ^b^	0.594
	no, low	0.032	0.083
	yes, high	0.375	0.212
	yes, low	0.206	0.111
*X*4	**Persistently Infected (PI) Group**		
	no BVDV in cohort^d^	0.236	0.259
	PI animal before cohort	0.103	0.010
	PI animal in cohort	0.394	0.370
	TI but no PI in cohort	0.266	0.361
*X*5	**BVDV vaccination**		
	no	0.879	0.915
	yes	0.121	0.085

^a^ Mean (standard deviation)

^b^ Proportion of each level

**Table 3:** Parameter estimates from the outcome and random feedlot effect exposure models.

Model	Parameters	Estimates of ***β***	Std. Error	z value	Pr(>|*Z*|)
Outcome Model	Intercept	1.2941	0.9672	1.3380	0.1809
	BVDV Induction	-0.5239	0.1345	-3.8957	0.0001
	Age	0.0988	0.1643	0.6010	0.5479
	Weight	-0.3572	0.1884	-1.8957	0.0580
	Mix History—no, low	-0.3967	0.3133	-1.2663	0.2054
	Mix History—yes, high	-0.2874	0.1505	-1.9099	0.0562
	Mix History—yes, low	-1.6589	0.2948	-5.6271	0.0000
	PI Group—PI animal before cohort	0.8575	0.3097	2.7687	0.0056
	PI Group—PI animal in cohort	0.2594	0.2213	1.1720	0.2412
	PI Group—TI but no PI in cohort	0.2243	0.2331	0.9624	0.3358
	BVDV Vaccination	0.7712	0.2252	3.4243	0.0006
Exposure Model	(Intercept)	1.3359	0.8449	1.5810	0.1139
	Age	-0.1830	0.1540	-1.1883	0.2347
	Weight	-0.3368	0.1733	-1.9430	0.0520
	Mix History—no, low	-0.9691	0.3291	-2.9449	0.0032
	Mix History –yes, high	0.8741	0.1505	5.8099	0.0000
	Mix History –yes, low	1.2922	0.2710	4.7687	0.0000
	PI Group—PI animal before cohort	3.0084	0.4798	6.2699	0.0000
	PI Group—PI animal in cohort	0.7692	0.1954	3.9375	0.0001
	PI Group—TI but no PI in cohort	0.3016	0.2007	1.5029	0.1329
	BVDV Vaccination	0.0155	0.2239	0.0693	0.9447

The risk of BRD with and without BVDV exposure from the Subset-BRD dataset, *P*_0_ and *P*_1_, were estimated by six different approaches (Approaches overview) as shown in Table 4. For each model, the estimated odds ratios were also obtained based on $\frac{{\hat{P}}_{1}/\left(1-{\hat{P}}_{1}\right)}{{\hat{P}}_{0}/\left(1-{\hat{P}}_{0}\right)}$. The standard error corresponding to each point estimate was obtained based on 500 bootstrap replicates from stratified sampling within each feedlot. Note that the most recognizable estimator of the conditional odds ratio (*exp*(*β*)) is not reported. That approach to estimation of the conditional odds ratio for the MOM would result in an odds ratio estimate of 0.5922 or *exp*(−0.5239). Using the *expit* to calculate the risk of the BRD under each exposure level in the MOM, and then estimating odds ratios based on ${\hat{P}}_{0}$ and ${\hat{P}}_{1}$ results in an OR of $\frac{0.4700/(1-0.4700)}{0.6513/(1-0.6513)}=0.4748$, where ${\hat{p}}_{0}=\frac{1}{N}\sum_{i=1}^{I}\sum_{j=1}^{J}expit\left({\hat{\beta }}_{a}+\sum_{p=1}^{k}{\hat{\beta }}_{p}X_{ij,p}+{\hat{U}}_{iY}\right)$ and ${\hat{p}}_{1}=\frac{1}{N}\sum_{i=1}^{I}\sum_{j=1}^{J}expit\left(\sum_{p=1}^{k}{\hat{\beta }}_{p}X_{ij,p}+{\hat{U}}_{iY}\right)$.

**Table 4:** The result of six approaches to estimate the effect of prior exposure to Bovine Viral Diarrhea Virus (BVDV) on Bovine Respiratory Disease (BRD) incidence using a subset of the NBRDI data with 1,552 cattle nested in 10 feedlots.

	${\hat{P}}_{0}$	se(${\hat{P}}_{0}$)	${\hat{P}}_{1}$	se(${\hat{P}}_{1}$)	$\hat{OR}$	se($\hat{OR}$)
UOM	0.6510	0.0199	0.4703	0.0143	0.4759	0.0520
MOM	0.6513	0.0199	0.4700	0.0143	0.4748	0.0521
IPW	0.6518	0.0532	0.5016	0.0148	0.5375	0.1207
ICPW	0.6490	0.0535	0.5083	0.0150	0.5591	0.1247
DR-IPW	0.6303	0.0339	0.5056	0.0144	0.6000	0.0931
DR-ICPW	0.6290	0.0339	0.5089	0.0145	0.6114	0.0946

^a^ UOM: Univariable outcome model; MOM: Multivariable outcome model

^b^ IPW: Inverse probability weighting; ICPW: Inverse conditional probability weighting

^c^ DR-IPW: Doubly robust with IPW; DR-ICPW: Doubly robust with ICPW

The outcome model approach includes “univariable outcome model (UOM)” and “multivariable outcome model (MOM)”. The MOM is a commonly used approach to modelling risk factor data such as these and is often employed in veterinary epidemiology. The weighting approach includes “IPW” and “ICPW”, where the “IPW” fits a random feedlot effect exposure model and weights each individual using the inverse probability of exposure. The “ICPW” fits a conditional logistic regression with feedlot as the stratum, and the weight for each individual is calculated using the inverse probability of exposure conditional on the covariates and sufficient statistic for the feedlot effect. The doubly robust approaches include both “DR-IPW” and “DR-ICPW”. The outcome models for both doubly robust approaches are the same as the multivariable outcome model, and the exposure model components are different (IPW and ICPW).

What is apparent from evaluation of the results in Table 4 is that the different estimation approaches provided quite different estimates of the marginal odds ratio. The UOM and MOM are methods that use conditional exchangeability in subsets defined by the confounding variables to estimate the effect of prior BVDV exposure on BRD in those subsets only, while instead we are interested in the marginal causal effect which can be quantified as the marginal odds ratio. If we limit our discussion to the estimate from the MOM model compared to the estimates from the weighting for the doubly robust methods, we see different average causal effects estimated from the models. The difference in estimates is apparent in *P*_0_ and *P*_1_. The analysis of the Subset-BRD data provided evidence of differences. However, because the true odds ratio is unknown, it is impossible to know which approach is the least biased. Therefore, we used simulation where the true values of the parameters are known, to illustrate that both the weighting and doubly robust approaches have advantage over the outcome models with reduced bias.

### Simulation study

Consistent with the objective of the manuscript, to document advantage of the weighting and doubly robust methods, here we present the simulation study and the performance of the six approaches used to analyze the Subset-BRD data. There are four different simulation scenarios. Under each scenario, we simulated the potential outcome $Y_{ij}^{a}$, exposure *A*_*ij*_, covariates ***X***_*ij*_ and feedlot ID. The observed outcome, *Y*_*ij*_, can be calculated from the potential outcome and exposure as $Y_{ij}=A_{ij}Y_{ij}^{a=1}+\left(1-A_{ij}\right)Y_{ij}^{a=0}$. Let *P*_*a*_ be the averaged potential outcome probabilities when *A*_*ij*_ = *a* for all samples. $P_{a}=\frac{1}{N}\sum_{i=1}^{I}\sum_{j=1}^{n_{i}}P\left(Y_{ij}^{a}=1\right)$, for *a* = 0 or 1. For each simulated dataset, we treated *P*_*a*_ as the true outcome probability under exposure *A*_*ij*_ = *a*. The true odds ratio (OR) was calculated as $OR=\frac{P_{1}/\left(1-P_{1}\right)}{P_{0}/\left(1-P_{0}\right)}$. For each approach, we calculated the average bias and root mean square error (RMSE) for each point estimate over 100 simulated datasets. The average true *P*_0_, *P*_1_ and odds ratio for 100 simulated datasets were also reported for comparison. In the following sections, we compare the performance among the outcome model approach, the weighting and the doubly robust approaches, and as well as the approaches within each approach-category.

#### Scenario 1

The goal for scenario 1 was to create a simulation setting that mimics the realistic data structure so the pattern of the realistic data estimates observed in Table 4 could be explained and compared with the true outcome probability *P*_*a*_. This dataset includes cluster (feedlot) sizes that are the same as the Subset-BRD data, which is not balanced and range from 16 to 418 animals.

In this simulation, the value of the covariates (age, weight, mix history, and PI group) ***X***_*ij*_ were directly taken from the Subset-BRD dataset. ${\hat{U}}_{iY}$ and ${\hat{U}}_{iA}$ were the random effects estimated from the outcome model and exposure model shown in Eqs 1 and 2 respectively. We then assigned exposure to the study units, and the exposure assignment mechanism was $P\left(A_{ij}=1\right)=expit\left(\sum_{p=1}^{k}{\hat{\alpha }}_{p}X_{ij,p}+{\hat{U}}_{iA}\right)$, where $\hat{\alpha }=\left({\hat{\alpha }}_{1},{\hat{\alpha }}_{2},...{\hat{\alpha }}_{5}\right)$ were the parameters estimated in the random effect exposure model shown in Table 3. For each animal, there were two potential outcomes generated, $Y_{ij}^{0}$ and $Y_{ij}^{1}$, where $P\left(Y_{ij}^{0}=1\right)=expit\left(\sum_{p=1}^{k}{\hat{\beta }}_{p}X_{ij,p}+{\hat{U}}_{iY}\right)$, and $P\left(Y_{ij}^{1}=1\right)=expit\left({\hat{\beta }}_{a}+\sum_{p=1}^{k}{\hat{\beta }}_{p}X_{ij,p}+{\hat{U}}_{iY}\right)$, where $\hat{\beta }=\left({\hat{\beta }}_{a},{\hat{\beta }}_{1},{\hat{\beta }}_{2},...,{\hat{\beta }}_{5}\right)$ were the parameters estimated from the outcome model shown in Table 3. The total sample size is 1552, the same as the Subset-BRD dataset. The result of scenario 1 is shown in Table 5. What we see in Table 5 is that the mean of the true risk of disease in the exposed animals over 100 simulated datasets is 0.5007 and 0.5931 for the unexposed animals. The mean of the true odds ratio for the 100 simulated datasets is 0.6890. We can also see that both the weighting and doubly robust approaches have smaller bias than the outcome model approach, and overall doubly robust approaches perform the best. This illustrates the advantage of the causal inference approaches in estimation of a marginal causal effect.

**Table 5:** Result of scenario 1, a simulation setting that mimics the realistic data, i.e. Subset-BRD dataset.

	*P*_0_	RMSE(${\hat{P}}_{0}$)	bias(${\hat{P}}_{0}$)	*P*_1_	RMSE(${\hat{P}}_{1}$)	bias(${\hat{P}}_{1}$)	*OR*	RMSE($\hat{OR}$)	bias($\hat{OR}$)
True	0.5931			0.5007			0.6890		
UOM	0.6462	0.0557	0.0530	0.4711	0.0308	-0.0296	0.4902	0.2039	-0.1988
MOM	0.6464	0.0559	0.0533	0.4708	0.0311	-0.0298	0.4893	0.2048	-0.1998
IPW	0.5904	0.0284	-0.0027	0.4998	0.0098	-0.0009	0.6980	0.0792	0.0089
ICPW	0.5901	0.0285	-0.0030	0.5030	0.0105	0.0023	0.7081	0.0825	0.0191
DR-IPW	0.5909	0.0228	-0.0022	0.5025	0.0094	0.0018	0.7030	0.0681	0.0140
DR-ICPW	0.5901	0.0230	-0.0030	0.5028	0.0097	0.0021	0.7064	0.0698	0.0174

#### Scenario 2

In Scenario 1, the cluster size was unbalanced. However, it might be of interest to know if the outcome model approach performs better when clusters are balanced. The purpose of the scenario 2 is then to evaluate all approaches under a balanced cluster (feedlot) size setting, where each feedlot has the same number of animals. We ensure the simulated datasets in this scenario to be close to the Subset-BRD dataset in size by sampling 155 animal ID’s from each of the 10 feedlots with replacement. Then we matched the corresponding covariates ***X***_*ij*_ and the estimated ${\hat{U}}_{iA}$ and ${\hat{U}}_{iY}$ from the Subset-BRD data. The exposure variable, *A*_*ij*_, and potential outcomes, $Y_{ij}^{0}$ and $Y_{ij}^{1}$, were also simulated following the same mechanism as scenario 1. The total sample size is 1550. The result of scenario 2 is shown in Table 6. Again, the advantage of weighting and doubly robust approaches is evident even when the cluster size is balanced. The outcome model approach has an estimated OR of 0.4098, while the true OR is 0.711, and the closest other method is DR-ICPW with 0.7342.

**Table 6:** Result of scenario 2, a balanced number of animals per feedlot setting.

	*P*_0_	RMSE(${\hat{P}}_{0}$)	bias(${\hat{P}}_{0}$)	*P*_1_	RMSE(${\hat{P}}_{1}$)	bias(${\hat{P}}_{1}$)	*OR*	RMSE($\hat{OR}$)	bias($\hat{OR}$)
True	0.4717			0.3882			0.7118		
UOM	0.5597	0.0892	0.0880	0.3419	0.0477	-0.0462	0.4106	0.3036	-0.3011
MOM	0.5600	0.0895	0.0883	0.3417	0.0479	-0.0465	0.4098	0.3044	-0.3019
IPW	0.4669	0.0280	-0.0048	0.3870	0.0117	-0.0012	0.7283	0.0902	0.0165
ICPW	0.4676	0.0279	-0.0041	0.3903	0.0120	0.0021	0.7365	0.0932	0.0247
DR-IPW	0.4677	0.0261	-0.0040	0.3898	0.0119	0.0016	0.7342	0.0881	0.0224
DR-ICPW	0.4676	0.0259	-0.0041	0.3899	0.0120	0.0018	0.7348	0.0872	0.0230

#### Scenario 3

In scenario 3, the aim was to evaluate the performance of these approaches in a more general setting to show that the patterns of estimation among the approaches were not caused by the choice of covariates or random effects. Instead of taking ***X***_*ij*_ or estimating *U*_*iA*_ and *U*_*iY*_ directly from the Subset-BRD data, we simulated these values according to the distributions in the Subset-BRD data. Table 7 shows the detailed distribution information about simulated ***X***_*ij*_, where the parameter values were chosen based on the covariate distributions in the Subset-BRD data. The feedlot-level random effect in the exposure model is $U_{iA}∼N\left(0,\sigma_{A}^{2}\right)$. The feedlot-level random effect in the outcome model is $U_{iY}∼N\left(0,\sigma_{Y}^{2}\right)$. Both *σ*_*A*_ and *σ*_*Y*_ can be estimated by the standard deviation of the random effects in the exposure model and outcome model fitted with the Subset-BRD data. Again, *A*_*ij*_, $Y_{ij}^{1}$ and $Y_{ij}^{0}$ followed the same simulation assignment mechanism as in scenario 1. The total sample size is 1550. The result of scenario 3 is shown in Table 8. We do not devote much text to the result of the simulation, as it is consistent with the prior scenarios, the least bias estimates are associated with doubly robust estimation methods, although the bias in the outcome regression models is less than in the two prior simulation scenarios.

**Table 7:** Distribution of covariates.

	Variable	distribution	parameter values
X1	Age	Normal	*μ*_1_ = 1.914, *σ*_1_ = 0.4183
X2	Weight	Normal	*μ*_2_ = 4.375, *σ*_2_ = 0.3630
X3	Mix History	Multinomial	*p*_1_ = 0.4639, *p*_2_ = 0.0509
			*p*_3_ = 0.3144, *p*_4_ = 0.1707
X4	PI Group	Multinomial	*p*_1_ = 0.2442, *p*_2_ = 0.0689
			*p*_3_ = 0.3853, *p*_4_ = 0.3015
X5	BVDV Vaccination	Binary	*p* = 0.1076
*U*_*A*_	Random effect in exposure model	Normal	*μ* = 0, *σ*_*A*_ = 0.7538
*U*_*Y*_	Random effect in outcome model	Normal	*μ* = 0, *σ*_*Y*_ = 1.2022

**Table 8:** Result of scenario 3, a general simulation setting.

	*P*_0_	RMSE(${\hat{P}}_{0}$)	bias(${\hat{P}}_{0}$)	*P*_1_	RMSE(${\hat{P}}_{1}$)	bias(${\hat{P}}_{1}$)	*OR*	RMSE($\hat{OR}$)	bias($\hat{OR}$)
True	0.4632			0.3685			0.6735		
UOM	0.4736	0.0293	0.0104	0.3624	0.0194	-0.0061	0.6373	0.1163	-0.0362
MOM	0.4739	0.0294	0.0107	0.3622	0.0195	-0.0063	0.6359	0.1167	-0.0376
IPW	0.4544	0.0321	-0.0088	0.3655	0.0139	-0.0030	0.6953	0.0997	0.0218
ICPW	0.4592	0.0321	-0.0040	0.3675	0.0139	-0.0010	0.6882	0.0992	0.0147
DR-IPW	0.4627	0.0259	-0.0004	0.3672	0.0122	-0.0013	0.6748	0.0792	0.0013
DR-ICPW	0.4627	0.0268	-0.0005	0.3672	0.0124	-0.0013	0.6751	0.0811	0.0016

#### Scenario 4

Scenario 4 is very similar to scenario 3 changing only the distribution of the PI group (X4) variable in order to have a stronger confounding variable. From the parameter estimates and the corresponding p-values in Table 9, we can see that PI group variable is contributing to both the outcome and random feedlot effect exposure model significantly. PI group follows a multinomial distribution with 4 levels, and the estimates of the first two levels are quite different from the remaining levels. In scenario 3, we used the original distribution of the PI group in the Subset-BRD data, in which the proportions of having PI group level 1 and level 2 are quite small. Now in scenario 4 we simulated PI group from a multinomial with *p*_1_ = 0.4, *p*_2_ = 0.4, *p*_3_ = 0.1, *p*_4_ = 0.1 by increasing the proportions of level 1 and level 2 to make PI group to be a stronger confounder. The result of scenario 4 is shown in Table 10. Again, the advantage of the weighting approach, and in particular with the doubly robust approach is evident, with the least bias associated with DR-IPW.

**Table 9:** Distribution of PI Group.

	Variable	distribution	parameter values
Old	PI Group	Multinomial	*p*_1_ = 0.2442, *p*_2_ = 0.0689
			*p*_3_ = 0.3853, *p*_4_ = 0.3015
New	PI Group	Multinomial	*p*_1_ = 0.4, *p*_2_ = 0.4
			*p*_3_ = 0.1, *p*_4_ = 0.1

**Table 10:** Result of scenario 4, a general simulation setting with stronger confounders.

	*P*_0_	RMSE(${\hat{P}}_{0}$)	bias(${\hat{P}}_{0}$)	*P*_1_	RMSE(${\hat{P}}_{1}$)	bias(${\hat{P}}_{1}$)	*OR*	RMSE($\hat{OR}$)	bias($\hat{OR}$)
True	0.4989			0.4035			0.6755		
UOM	0.4734	0.0431	-0.0255	0.4158	0.0193	0.0123	0.8044	0.2051	0.1289
MOM	0.4735	0.0430	-0.0254	0.4157	0.0193	0.0122	0.8036	0.2047	0.1281
IPW	0.4861	0.0500	-0.0128	0.4015	0.0101	-0.0021	0.7225	0.1638	0.0470
ICPW	0.4936	0.0512	-0.0053	0.4035	0.0102	0.0000	0.7081	0.1637	0.0326
DR-IPW	0.4961	0.0382	-0.0028	0.4033	0.0092	-0.0002	0.6929	0.1209	0.0174
DR-ICPW	0.4962	0.0399	-0.0026	0.4034	0.0094	-0.0001	0.6932	0.1259	0.0177

#### Doubly robust sensitivity analysis

One of the anticipated difficulties associated with doubly robust approach is the need to assess the different impact from the misspecified model of the exposure used to estimate the inverse probability weight or the misspecified outcome model used to estimate the *m*_0_, *m*_1_. By “misspecified model” we mean changing the link function we used in the logistic regressions (e.g. logit). Therefore, to assess the performance of doubly robust approaches and compare them with the other approaches under the one-model-misspecification situation, we performed a sensitivity analysis following the setting in scenario 3 by deliberately misspecifying the model.

To create the outcome model misspecification setting, we used a logit link for the outcome model, but $Y_{ij}^{0}$ and $Y_{ij}^{1}$ were actually simulated from binary distributions with cloglog link function. Similarly, to create the exposure model misspecification setting, we used a logit link for the exposure model, but *A*_*ij*_ was actually simulated from a binary distribution with cloglog link function. Tables 11 and 12 show the results of all approaches under the outcome model misspecification situation and the exposure model misspecification situation respectively.

**Table 11:** Result of the doubly robust sensitivity analysis under outcome model misspecification.

	*P*_0_	RMSE(${\hat{P}}_{0}$)	bias(${\hat{P}}_{0}$)	*P*_1_	RMSE(${\hat{P}}_{1}$)	bias(${\hat{P}}_{1}$)	*OR*	RMSE($\hat{OR}$)	bias($\hat{OR}$)
True	0.5678			0.4522			0.6226		
UOM	0.5893	0.0377	0.0215	0.4396	0.0250	-0.0126	0.5488	0.1378	-0.0738
MOM	0.5897	0.0379	0.0219	0.4393	0.0251	-0.0128	0.5473	0.1384	-0.0753
IPW	0.5616	0.0281	-0.0062	0.4473	0.0124	-0.0049	0.6304	0.0818	0.0078
ICPW	0.5674	0.0292	-0.0004	0.4500	0.0119	-0.0021	0.6231	0.0851	0.0005
DR-IPW	0.5681	0.0221	0.0003	0.4501	0.0097	-0.0021	0.6192	0.0641	-0.0033
DR-ICPW	0.5680	0.0230	0.0002	0.4501	0.0098	-0.0021	0.6197	0.0663	-0.0029

**Table 12:** Result of the doubly robust sensitivity analysis under exposure model misspecification.

	*P*_0_	RMSE(${\hat{P}}_{0}$)	bias(${\hat{P}}_{0}$)	*P*_1_	RMSE(${\hat{P}}_{1}$)	bias(${\hat{P}}_{1}$)	*OR*	RMSE($\hat{OR}$)	bias($\hat{OR}$)
True	0.4633			0.3676			0.6690		
UOM	0.4838	0.0515	0.0205	0.3608	0.0173	-0.0068	0.6188	0.1766	-0.0503
MOM	0.4842	0.0518	0.0209	0.3606	0.0174	-0.0070	0.6173	0.1770	-0.0517
IPW	0.3656	0.1063	-0.0977	0.3733	0.0153	0.0056	1.0549	0.4344	0.3858
ICPW	0.3719	0.1008	-0.0914	0.3754	0.0171	0.0078	1.0356	0.4151	0.3665
DR-IPW	0.4611	0.0269	-0.0022	0.3669	0.0118	-0.0007	0.6788	0.0841	0.0098
DR-ICPW	0.4612	0.0285	-0.0021	0.3669	0.0121	-0.0007	0.6789	0.0873	0.0099

### Summary discussion of simulations results

All the scenarios indicate that the doubly robust approaches have the best performance for consistency and stability overall, where the difference between DR-IPW and DR-ICPW are very small. The sensitivity analysis result suggests that the doubly robust approaches also have the best performance when either the outcome model or the exposure model are incorrectly specified. When the outcome model is incorrectly specified, the performance of the weighting approach should not be influenced. However, in Table 11 the RMSE’s for the doubly robust approaches are still smaller than the weighting approach. Similarly in Table 12, the doubly robust approaches outperform the weighting approaches when the exposure model is incorrectly specified.

## Discussion and conclusions

In this paper, we aimed to document the advantages of using weighting and doubly robust approaches to estimate the average causal effect of an exposure on the outcome. Our rationale was that although the methods have been available for decades, these approaches, which are less biased, appear to be infrequently used in veterinary epidemiology. If the goal of research is to obtain the least biased estimation of causal effect, then it seems reasonable that epidemiologists will employ methods as suggested. The approaches we recommended here have been documented previously [18, 23, 24], although perhaps not so explicitly with realistic veterinary data.

In an observational study, IPW, ICPW and doubly robust estimation are useful in estimating the causal effect of the exposure when there are confounders involved. IPW adjusts for confounders by creating a pseudo-population where the measured confounders and exposure are independent. ICPW is robust for clustered data when the cluster-level confounders are not measured. Doubly robust estimation is a combination of traditional outcome model and exposure model (IPW or ICPW) idea, which provides stable and consistent estimates if at least one of the outcome model or exposure model are correctly specified. As the true relationship among exposure, outcome, and confounders are rarely known, the doubly robust estimation has the advantage in both stability and consistency in estimation.

When compared to the traditional outcome model approach in the application to the NBRDI data, the results from IPW, ICPW and doubly robust estimation showed considerable amount of difference in the estimated effect of the exposure on outcome. Simulation studies mimicking the Subset-BRD dataset revealed that the IPW, ICPW and doubly robust estimation methods are superior to the traditional outcome model approach in both bias and precision of estimation. Further simulation studies showed that the doubly robust methods are robust to model misspecification and is thus the recommended approach.

## Supporting information

S1 File R code. R code for functions and Subset-BRD data analysis.
(R) Click here for additional data file.

S2 File Search. Search conducted on July 8th, 2019 using PubMed interface.
(TXT)

S3 File ICPW. Assumptions and details for ICPW approach.
(PDF)

S4 File Dataset. De-identified dataset in csv format.
(CSV)
