# Supplementary material for: Comparing the estimates of effect obtained from statistical causal inference methods: An example using bovine respiratory disease in feedlot cattle
Source: PLoS One. 2020 Jun 25;15(6):e0233960. doi: 10.1371/journal.pone.0233960 (PMC7316239; doi:10.1371/journal.pone.0233960)
Supplement: S3 File — Assumptions and details for ICPW approach. (PDF) [file pone.0233960.s003.pdf]

Assumptions and ICPW details

Assumptions of ICPW

1. For each cluster, there exists a function of $\boldsymbol{A}_i$, $S_i = S_i(\boldsymbol{A}_i)$, which is sufficient for V_i in the exposure model. Moreover, for any value s in S_i and any unit j, there exist at least two different possible values of A_{ij} in $\boldsymbol{A}_i$, i.e. a_{ij} in $\boldsymbol{a}_i$ and a^*_{ij} in $\boldsymbol{a}^*_i$, such that i) $a_{ij} \neq a^*_{ij}$ and ii) $S_i(\boldsymbol{a}_i) = S_i(\boldsymbol{a}^*_i) = s$.

2. *Cluster-level exchangeability:* $\{\boldsymbol{Y}^0_i, \boldsymbol{Y}^1_i\} \amalg \boldsymbol{A}_i | \boldsymbol{X}_i, S_i$ *for all* i.

3. *Cluster-level positivity: For all* i, $\sum_{j=1}^{n_i} a_{ij} \neq 0$ *or* n_i, *and* $0 < P(A_{ij} = a_{ij} | \boldsymbol{X}_i, S_i = S(\boldsymbol{a}_i)) < 1$ *for all* i *and* j, *and* $\boldsymbol{a}_i = (a_{i1}, a_{i2}, ..., a_{in_i})$.

ICPW estimator

The major difference between the IPW and ICPW is that we construct the probability of exposure (the propensity score) A_{ij} conditional on individual-level covariates $\boldsymbol{X}_{ij}$ and the sufficient statistic of the cluster-level covariates V_i (denoted as $S_i = S(\boldsymbol{A}_i)$), which is a function of $\boldsymbol{A}_i = (A_{i1}, A_{i2}, ..., A_{in_i})$. This is done using a conditional logistic regression for the exposure model. Formally, this is as follows in Eq 1, let $\boldsymbol{a}_i$ be the observed value of $\boldsymbol{A}_i$, the exposure model for ICPW estimator can be fitted with a conditional logistic regression as follows:

$$\begin{aligned} P(A_{ij} = a_{ij} | \boldsymbol{X}_{ij}, S_i; \boldsymbol{\alpha}) &= \frac{P(A_{ij} = a_{ij}, S_i = S(\boldsymbol{a}_i) | \boldsymbol{X}_{ij}, V_i; \boldsymbol{\alpha})}{P(S_i = S(\boldsymbol{a}_i) | \boldsymbol{X}_{ij}, V_i; \boldsymbol{\alpha})} \\ &= \frac{\sum_{a^* \in \Omega_{i,j}} P(\boldsymbol{A}_i = \boldsymbol{a}^*_i | \boldsymbol{X}_{ij}, V_i; \boldsymbol{\alpha})}{\sum_{\tilde{\boldsymbol{a}} \in \tilde{\Omega}_i} P(\boldsymbol{A}_i = \tilde{\boldsymbol{a}}_i | \boldsymbol{X}_{ij}, V_i; \boldsymbol{\alpha})}, \end{aligned} \tag{1}$$

for all i, j, and any value $\boldsymbol{a}_i \in \Omega_i$. Also $\Omega_{i,j} = \{\boldsymbol{a}^* \in \Omega_i | a^*_j = a_{ij} \text{ and } S_i(\boldsymbol{a}^*) = S_i(\boldsymbol{a}_i)\}$ and $\tilde{\Omega}_i = \{\tilde{\boldsymbol{a}} \in \Omega_i | S_i(\tilde{\boldsymbol{a}}) = S_i(\boldsymbol{a}_i)\}$.

For the ICPW estimator, we use $\hat{P}_{a,ICPW}$ in Eq 2 to estimate the risk of disease under exposure level $A = a$. For a binary exposure, A_{ij} the estimators are as follows:

$$\begin{aligned} \hat{P}_{1,ICPW} &= \frac{1}{N} \sum_{i=1}^{I} \sum_{j=1}^{n_i} \frac{A_{ij} Y_{ij}}{P(A_{ij} = 1 | \boldsymbol{X}_{ij}, S_i; \hat{\boldsymbol{\alpha}})}, \\ \hat{P}_{0,ICPW} &= \frac{1}{N} \sum_{i=1}^{I} \sum_{j=1}^{n_i} \frac{(1 - A_{ij}) Y_{ij}}{1 - P(A_{ij} = 1 | \boldsymbol{X}_{ij}, S_i; \hat{\boldsymbol{\alpha}})}. \end{aligned} \tag{2}$$

Let $\hat{\boldsymbol{\alpha}}$ be the conditional maximum likelihood estimator that maximizes the joint conditional likelihood $L^c(\boldsymbol{\alpha}) = \prod_{i=1}^{I} \prod_{j=1}^{n_i} P(A_{ij} = 1 | \boldsymbol{X}_{ij}, S_i; \boldsymbol{\alpha})$. Under the logistic model, the sufficient statistic of V_i is the exposure sum in the cluster, i.e. $S_i(\boldsymbol{A_i}) = \sum_{j=1}^{n_i} A_{ij}$.
